# Large neck metastasis of hypopharyngeal cancer

**DOI:** 10.1002/ccr3.2503

**Published:** 2019-11-23

**Authors:** Massimo Ralli, Arianna Di Stadio, Marco De Vincentiis, Antonio Greco

**Affiliations:** ^1^ Department of Sense Organs Sapienza University of Rome Rome Italy; ^2^ Department of Otolaryngology University of Perugia Perugia Italy; ^3^ Department of Oral and Maxillofacial Sciences Sapienza University of Rome Rome Italy

**Keywords:** cancer, magnetic resonance imaging, Oncology

## Abstract

Lateral neck masses may be due to congenital, infectious, or malignant disease. Sometimes, the mass can be the only sign of an asymptomatic cancer. A correct clinical approach and the differential diagnosis are the key points for a correct diagnosis.

## Graphical abstract

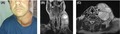

A 55‐year‐old man presented with a 3‐month history of dysphagia and swelling in the left side of the neck. The patient had been previously treated with antibiotics and corticosteroids, but the mass continued to be worsen. Clinical examination revealed a large solid neck mass, painful at palpation (Figure 1 A).

**Figure 1:**
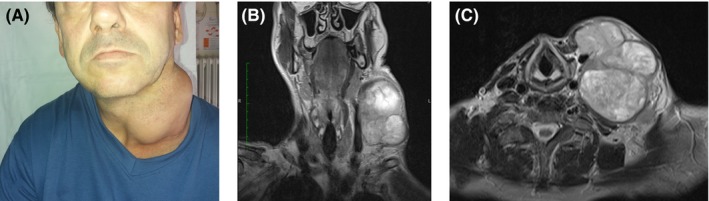
(A) A large solid mass is seen in the left portion of the neck. Coronal (B) and axial (C) contrast‐enhanced magnetic resonance imaging scan showing a T1‐hyperintense solid neck mass

A contrast‐enhanced magnetic resonance imaging (MRI) demonstrated a T1‐hyperintense, solid, homogeneous mass in the left side of the neck measuring 102 × 94 × 108 mm and compressing the left internal jugular vein (Figure 1 B and C); no additional alterations were found in head and neck imaging. Endoscopic examination of the upper airways showed an exophytic tissue in the left pyriform sinus. A biopsy of the hypopharyngeal mass was performed; histology was consistent with HPV‐negative (clone 4c4) lymphnode metastasis of hypopharyngeal squamous cell carcinoma (HPSCC) (phenotype: CKAE1AE3+, p63+, CKHMW+, CK7‐, and CK20‐). HPSCC represents about 7% of all cancers of the upper aerodigestive tract and is the one with the worst prognosis, with 5‐year survival rates for stage IV near 24%.^1^ Delayed diagnosis, common due to the lack of early‐stage symptoms, contributes to poor prognosis.^2^ Asymptomatic neck masses may represent the initial or, in some cases, the only clinical manifestation of hypopharyngeal cancer; therefore, timely identification of primary tumor is of paramount importance.

## CONFLICT OF INTERESTS

The authors report no competing interests.

## AUTHOR CONTRIBUTIONS

Dr MR: collected the data, analyzed the results, and wrote the article Dr ADS: supported in the article writing, analyzed the results, presented criticism to the paper, and prepared the figures. Professor MDV: collected the data, analyzed the results, and supervised the writing. Professor AG: collaborated in data collection, analyzed the results, and supervised the work.
